# Prevalence of Obesity in Carpal Tunnel Syndrome Patients: A Cross-Sectional Survey

**DOI:** 10.7759/cureus.1519

**Published:** 2017-07-26

**Authors:** Salman Mansoor, Maimoona Siddiqui, Farrukh Mateen, Shoab Saadat, Zarak H Khan, Mehr Zahid, Hamza H Khan, Shuja A Malik, Salman Assad

**Affiliations:** 1 Neurology, Registrar Neurology, Cork University Hospital; 2 Consultant Neurologist, Department of Neurology, Shifa International Hospital, Islamabad, Pakistan; 3 Department of Neurology, Shifa International Hospital, Islamabad, Pakistan; 4 Department of Nephrology, Shifa International Hospital, Islamabad, Pakistan; 5 Department of Medicine, Shifa College Of Medicine; 6 Internal Medicine, University of Lahore, Lahore, Pakistan; 7 Graduate, Shifa International Hospital, Islamabad, Pakistan; 8 Internal Medicine, Nawaz Sharif Medical College, University of Gujrat; 9 Department of Medicine, Shifa International Hospital, Islamabad, Pakistan

**Keywords:** carpal tunnel syndrome, body mass index, obesity, pakistan

## Abstract

Carpal tunnel syndrome (CTS) is the most common compressive entrapment neuropathy caused by the compression of the median nerve at the wrist space known as the carpal tunnel. The epidemiologic factors related to CTS include genetic, medical, social, vocational, and demographic factors. The common symptoms experienced include pain, paresthesia, and numbness in the median nerve distribution. If left untreated, it can lead to irreversible median nerve damage, causing a loss of hand function. Body mass index (BMI) has been attributed as a risk factor for the development of CTS.

We planned to determine the frequency of obesity among CTS patients in the neurophysiology department of a tertiary care center in Islamabad, Pakistan. The survey was designed as a cross-sectional descriptive study from March 2016 to August 2016 using a consecutive nonprobability sampling technique. A total of 112 patients with a mean age of 54 ± 5 years were included in the study. In the study population, 39 patients (35 percent) were males and 73 were females (65 percent). Based on BMI, 74 patients (66 percent) had a normal weight and 38 (34 percent) were obese. The frequency of obesity in our study was 34 percent, excluding the other comorbid conditions, which is quite high. Targeted therapy in those with CTS should also include weight reduction measures because obesity poses a cause-and-effect relationship for both the severity and the pathogenesis of CTS.

## Introduction

Carpal tunnel syndrome (CTS) is the most common compressive entrapment neuropathy caused by the compression of the median nerve at a space around the wrist known as the carpal tunnel. The prevalence of CTS in the United Kingdom is 70-160 cases per 1,000 subjects [1-3]. Many risk factors have been correlated with the development of this compressive neuropathy. The damage usually starts as demyelination, which later progresses to axonal degeneration. The course of the disease first affects the sensory fibers followed by the motor fibers.

Body mass index (BMI) has been attributed as a risk factor for the development of CTS [4-6]. Obesity is also a modifiable risk factor with lifestyle modifications. It has been found that a recent gain in weight is a risk factor for CTS because of increased fluid accumulation in the tissue spaces in the carpal tunnel [7]. A statistically significant relationship was found between the first hospital referral and Quetelet’s obesity index [8]. A study looking at the risks for CTS found a statistically significant relationship with a two-fold risk for CTS in people who attend slimming courses [9]. There was a highly significant increase in the average BMI of female industrial workers presenting after five years of follow-up [10]. Another study showed a lower incidence of CTS in slender individuals (BMI less than 20) with a lower incidence of CTS (16 percent) as compared to the 39 percent in obese patients with an average BMI of more than 29 [11]. An obese individual had 2.5 times higher probability of CTS as compared to a slender individual with a mean BMI of 28.9 and 26.2, respectively. Interestingly, no correlation was found between obesity and ulnar sensory distal latencies, inferring that obesity affects nerves differently [12].

There is a dearth of knowledge and literature in our population that looks at this correlation or at any lifestyle modifications that might reverse this potentially treatable condition. Disagreement still exists on the correct and accurate diagnosis of CTS. However, most experts can agree that a combination of nerve conduction studies (NCS) along with the subjective symptoms are currently deemed the gold standard for diagnosis of CTS [13]. We designed this survey to look at the frequency of obesity among patients with CTS, which may serve to direct physicians in addressing obesity for a better outcome in treating CTS.

## Materials and methods

This was a descriptive cross-sectional study in the neurophysiology laboratory of a tertiary care hospital in Islamabad. Data were collected from 1 March 2016 to 31 August 2016 through consecutive nonprobability convenience sampling. We kept the prevalence rate at 39 percent for obese patients, with a 95 percent confidence interval; the sample size estimation by the World Health Organization (WHO) calculator was 112. Patients with an age range of 18-65 years, showing symptoms pertinent to CTS and fulfilling the electrophysiological criteria, participated. Patients with other confounding factors that may cause CTS were excluded. These conditions were screened based on past medical history: pregnancy, diabetes mellitus (DM), traumatic injury, inflammatory arthritis, amyloidosis, hypothyroidism, acromegaly, stroke, paraneoplastic conditions, rheumatoid arthritis, and those on exogenous corticosteroids and estrogens. Permission was taken from the ethical committee and institutional review board (IRB) of the hospital. The BMIs of all patients who were diagnosed with CTS on electrophysiological parameters, as described above, were calculated.

Nerve conduction studies

All the studies done in the neurophysiological department by neurophysiologists and that were interpreted and reported on by consultant neurologists were included in the study. Standard nerve conduction studies (NCS) were performed on CTS subjects using the digital Windows XP-based machine from Nihon Kohden (Tokyo, Japan), model number MEB -2200/MEB 9100. Motor and sensory studies were performed on both hands of every patient according to the guidelines prescribed by the American Academy of Electrodiagnostic Association (AAEDA).

Motor studies

To start with, motor studies were performed orthodromically, starting from the distal to the proximal nerve sites (from wrist to elbow), followed by sensory NCS, which were performed antidromically. The reference skin temperature for all patients was maintained at 35°C for motor studies started at the supramaximal intensity with a starting stimuli of a 0.2 m/sec duration, a standardized set of low-frequency filters (LFFs) of 2 Hz and high-frequency filters (HFFs) of 3 kHz, and a stimulation rate of 1 Hz. A sweep speed of 2 m/sec with a sampling time of 100 msec was set with a gain of 5-10 mV per division. The G1 electrode was set at the belly of the abductor pollicis brevis and the G2 electrode was placed at the tendon of the abductor pollicis brevis, at least 4 cm away from the G1 site. The earthing electrode was applied in between the G1 stimulation site at a more prominent bony site.

For the median nerve, a distal stimulation that was 7 cm proximal from the G1 electrode was given across the flexor retinaculum followed by another stimulation at the elbow median to the brachial artery site.

For the ulnar nerve, distal stimulation was given at 7 cm proximal to the G1 electrode placed at the belly of the abductor digiti minimi muscle. At the elbow, pre-elbow stimulation was given at a point just above the olecranon tuberosity. All waves recorded at different sites were recorded and marked from peak to peak and from takeoff to the resting wave points.

Individual and comparative analyses were done for the median to ulnar differences. The distal latency and compound motor action potential (CMAP) were recorded and calculated for further analysis.

Sensory studies

For the sensory nerve studies, wiring electrodes were applied from wrist to index finger for the median nerve and from wrist to little finger for the ulnar nerve. Stimulation was given with settings starting at an intensity of 0.5 mV, a sensitivity of 50mV, LFF of 2 Hz, HFF of 3 kHz, an analysis time of 2 msec per division, and a sampling time of 100 msec with a stimulation interval of 100 msec and a duration of 0.2 msec.

A starting stimulation of 1 mV intensity was given and slowly increased to get optimum reciprocal waves. The median and ulnar distances of 13 cm and 11 cm were set for stimulation, respectively. The waves that appeared were recorded and marked for latency with single nerve action potential values. Data were gathered for individual waves and the combined nerve difference in latency and amplitudes for further calculations.

Data were collected on the predesigned questionnaire. The data were then entered and analyzed using statistical package for social sciences (SPSS) version 21 (IBM, Chicago, IL) format; descriptive statistics were calculated. For qualitative variables, such as gender and frequencies, percentages for obese and normal-weight individuals were calculated. Effect modifiers, such as age, gender, and the severity of the disease, were controlled by stratification. A post-stratification chi-square (X2) was applied and a p-value of less than 0.05 was considered statistically significant.

## Results

**Figure 1:**
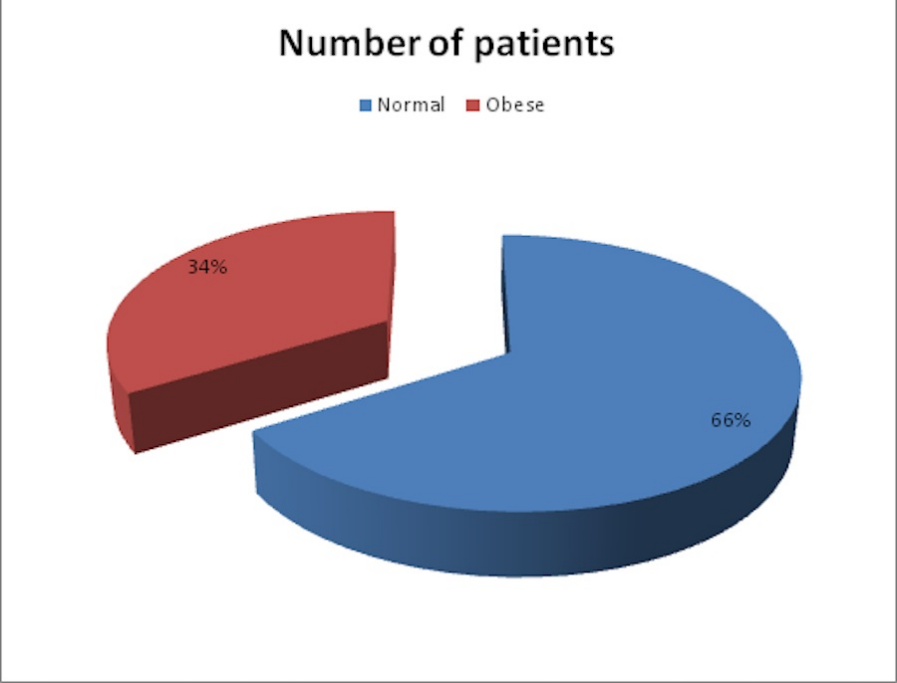
BMI distribution among patients

A total of 112 patients were enrolled in this study, with an age range of 38-63 years and a mean age of 54 ± 5 years. Out of the 112 patients, 2 patients (2 percent) were in the age group of 31-40 years, 24 patients (21 percent) were in the age group of 41-50 years, 75 patients (67 percent) were in the age group of 51-60 years, and 11 patients (10 percent) were in the age group of 61-65 years. In the study population, 39 patients (35 percent) were males and 73 (65 percent) were females. Based on the BMI, there were 74 patients (66 percent) of normal weight (BMI less than 25) and 38 (34 percent) were obese (BMI more than 30) (Figure 1).

**Figure 2:**
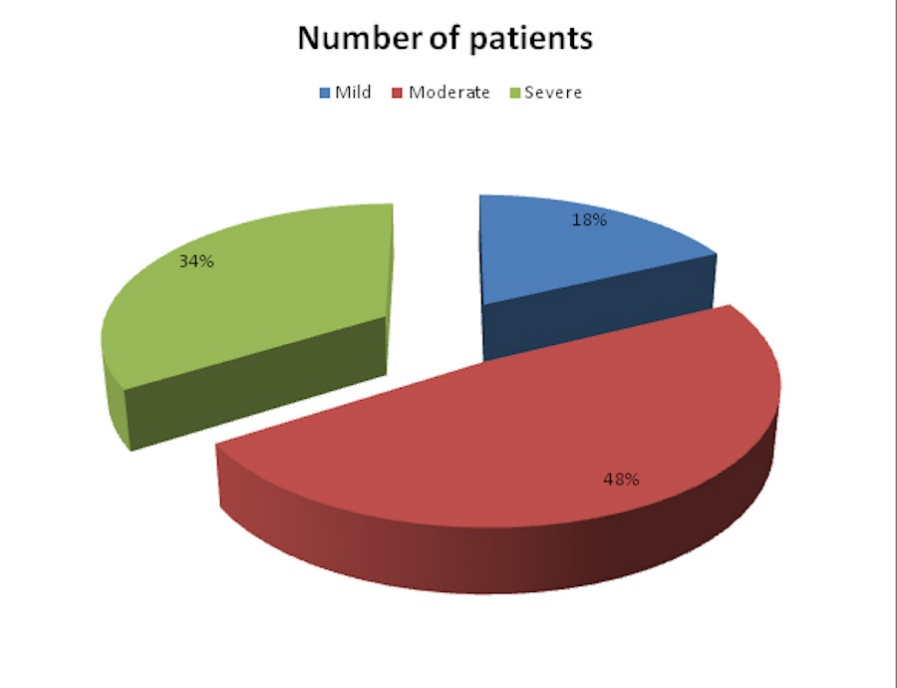
Categorization of patients based on the severity of disease

Out of 112 patients, 20 patients (18 percent) had mild CTS, 54 (48 percent) had moderate CTS, and 38 (33 percent) had severe CTS (Figure 2).

Out of 74 patients in the normal weight category, there were 30 males and 44 females. Out of the 38 patients in the obese category, there were 9 males and 29 females. The chi-square (*X2 ) *value was found to be not statistically significant and there was no association between patient gender and their BMI (p more than 0.05).

According to BMI in the normal weight category, out of 78 patients, there were 14 patients with mild CTS, 33 patients with moderate CTS, and 27 patients with severe CTS. The severity of CTS was assessed based on clinical symptoms and electrophysiological studies. In the obese category, there were 6 patients with mild CTS, 21 patients with moderate CTS, and 11 patients with severe CTS. There were no statistically significant results observed between BMI and the severity of CTS (p more than 0.05) (Table 1).

**Table 1:** Stratification of BMI based on the severity of CTS

		Severity of CTS			Total	P-Value
		Mild	Moderate	Severe		
BMI category of patients	Normal	14	33	27	74	0.562
	Obese	06	21	11	38	
Total		20	54	38	112	

## Discussion

In our study, 38 patients (34 percent) were obese. A case-control study by Becker J et al. documented 791 CTS cases and 981 controls. He reported that female gender, BMI more than 30, the age of 41–60 years, and diabetes mellitus were significantly more frequent in the case group. Males tend to have more severe CTS and diabetes mellitus was a significant risk factor for bilateral lesions. A stratified analysis showed female gender, obesity, and the age of 41–60 years as independent risk factors [14]. One possible explanation for this contrast in our study may be the smaller sample size. In a clinical review of patients who had undergone a surgical decompression of the median nerve in the carpal tunnel, results indicated that the three-year-period prevalence of CTS in females is more than that in males. Proportionally, there were more patients over the age of 55 years than in the general population. Similar results with a mean age of 53 and a standard deviation (SD) of 5.2 were observed in our survey.

It was also found that carpal tunnel release (CTR) patients are two times as likely to be overweight (BMI more than 25) than the general population and female patients are two times as likely to be obese (BMI more than 30) than the general population [15]. This trend was also observed in our survey with females being more obese than male patients, at 39 percent and 30 percent, respectively. In another study by Kurt S et al., the presence or absence of recovery in median nerve conduction velocities after weight loss in obese patients was assessed to determine whether excess weight or other factors influence the higher prevalence of CTS in obese patients. Patients with BMIs equal to or greater than 30 were included in the study. Nerve conduction studies (NCSs) were obtained on one upper extremity. All patients were included in dietetic programs. Three months later, NCSs were repeated and compared with the first NCS. BMIs were statistically significantly lower on the second visit three months later (p = 0.0001). There was a statistically significant difference seen in the second NCS of electromyographically diagnosed cases with CTS [16].

With its limited design, our survey was unable to find the outcomes if these patients were enrolled in weight control programs. However, it does raise the question for future researchers to identify the ideal weight-based targets for an effective CTS treatment regimen. In our study participants, the frequency of obesity was 33.9 percent, which is very high. A large case-control study using the UK general practice research database (GPRD) included 3,391 cases, of which 2,444 (72 percent) were women, with a mean age at diagnosis of 46 years (range 16–96). A multivariate analysis showed that the risk factors associated with CTS were previous wrist fracture, rheumatoid arthritis, osteoarthritis of the wrist, obesity, diabetes, and the use of insulin, sulphonylureas, metformin, and thyroxine [17]. Sedentary lifestyles, eating habits, or an unaddressed metabolic syndrome may be factors for such a high number of obesity cases in our country; this needs to be further explored. 

A meta-analysis by Shiri R et al. included 58 studies, consisting of 1,379,372 individuals. Overweight participants increased the risk of CTS or CTR 1.5 fold (pooled confounder-adjusted odds ratio (OR) = 1.47, 95% CI 1.37-1.57, N = 1,279,546) and obesity twofold (adjusted OR = 2.02, 95% CI 1.92-2.13, N = 1,362,207). Each one unit increase in BMI increased the risk of CTS by 7.4 percent (adjusted OR = 1.074, 95% CI 1.071-1.077, N = 1,258,578). Overweight and obesity had stronger effects on CTR than on CTS [18].

In an effort to determine the relative risk (RR) of obesity in the development of CTS, 949 patients who had an evaluation of the right upper extremity, which included motor and sensory conduction studies of the median and ulnar nerves, were reviewed. Of these patients, 261 were diagnosed with a median mononeuropathy at the wrist. Those individuals who were classified as obese (BMI more than 30) were 2.5 times more likely than slender individuals (BMI less than 20) to be diagnosed with CTS. A total of 43 percent of obese women and 32 percent of obese men had a diagnosis of CTS compared to 21 percent of slender women and 0 percent of slender men [11].

## Conclusions

CTS is a disease with significant morbidity, and it has been considered idiopathic in most cases. The frequency of obesity in our study was 34 percent, excluding the other comorbid conditions, which is fairly high. Targeted therapy in those with CTS should also include weight reduction measures because obesity poses a cause-and-effect relationship for both the severity and the pathogenesis of CTS. Large prospective trials looking at the effects of an effective obesity countering regimen should be employed to explore the optimal weight reduction parameters for CTS in our population.

## References

[1] Alemdar N (2015). Value of F-wave studies on the electrodiagnosis of carpal tunnel syndrome. Neuropsychiatr Dis Treat.

[2] Kozak A, Schedlbauer G, Wirth T (2015). Association between work-related biomechanical risk factors and the occurrence of carpal tunnel syndrome: an overview of systematic reviews and a meta-analysis of current research. BMC Musculoskelet Disord.

[3] Unno F, Lucchina S, Bosson D (2015). Immediate and durable clinical improvement in the non-operated hand after contralateral surgery for patients with bilateral Carpal Tunnel Syndrome. BMC Musculoskelet Disord.

[4] Zyluk A, Dabal L, Szlosser Z (2011). Association of anthropometric factors and predisposition to carpal tunnel syndrome. Chir Narzadow Ruchu Ortop Pol.

[5] Enhesari A, Saied A, Mohammadpoor L (2014). Presence or absence of palmaris longus and fifth superficial flexor digitorum; is there any effect on median nerve surface area in wrist sonography. Iran J Radiol.

[6] Vögelin E, Mészàros T, Schöni F (2014). Sonographic wrist measurements and detection of anatomical features in carpal tunnel syndrome. Scientific World J.

[7] Ghali J, Murugasu A, Day T (2012). Carpal tunnel syndrome in fabry disease. JIMD Rep.

[8] Coggon D, Ntani G, Harris EC (2013). Impact of carpal tunnel surgery according to pre-operative abnormality of sensory conduction in median nerve: a longitudinal study. BMC Musculoskelet Disord.

[9] Kim DH, Cho BM, Oh SM (2014). Delayed improvement after endoscopic carpal tunnel release. J Korean Neurosurg Soc.

[10] Ganeriwal A, Biswas D, Srivastava T (2013). The effects of working hours on nerve conduction test in computer operators. Malays Orthop J.

[11] Werner RA, Albers JW, Franzblau A (1994). The relationship between body mass index and the diagnosis of carpal tunnel syndrome. Muscle Nerve.

[12] Ebrahimzadeh MH, Mashhadinejad H, Moradi A (2013). Carpal tunnel release in diabetic and non-diabetic patients. Arch Bone Jt Surg.

[13] Oskouei AE, Talebi GA, Shakouri SK (2014). Effects of neuromobilization maneuver on clinical and electrophysiological measures of patients with carpal tunnel syndrome. J Phys Ther Sci.

[14] Becker J, Nora DB, Gomes I (2002). An evaluation of gender, obesity, age and diabetes mellitus as risk factors for carpal tunnel syndrome. Clin Neurophysiol.

[15] Lam N, Thurston A (1998). Association of obesity, gender, age and occupation with carpal tunnel syndrome. Aust N Z J Surg.

[16] Kurt S, Kisacik B, Kaplan Y (2008). Obesity and carpal tunnel syndrome: is there a causal relationship?. Eur Neurol.

[17] Geoghegan JM, Clark DI, Bainbridge LC (2004). Risk factors in carpal tunnel syndrome. J Hand Surg Br.

[18] Shiri R, Pourmemari MH, Falah-Hassani K (2015). The effect of excess body mass on the risk of carpal tunnel syndrome: a meta-analysis of 58 studies. Obes Rev.

